# Study of the Hypoglycemic Activity of Rhamnolipids Using the *In Ovo* Model

**DOI:** 10.3390/cimb48070664

**Published:** 2026-06-28

**Authors:** Margarida Queirós, Rute S. Moura, Eduardo J. Gudiña

**Affiliations:** 1 CEB—Centre of Biological Engineering, University of Minho, 4710-057 Braga, Portugal; 2 Life and Health Sciences Research Institute (ICVS), School of Medicine, University of Minho, 4710-057 Braga, Portugal; 3 ICVS/3B’s—PT Government Associate Laboratory, Braga, 4710-057 Guimarães, Portugal; 4 LABBELS—Associate Laboratory, Braga, 4710-057 Guimarães, Portugal

**Keywords:** biosurfactant, blood glucose, chicken embryo, diabetes, glucose transport proteins, insulin

## Abstract

The prevalence of diabetes has increased considerably in recent decades, representing a global health problem. Although several pharmacological approaches for the treatment of diabetes are available, it is pertinent to explore more effective alternatives with reduced adverse effects. In this work, the hypoglycemic activity of rhamnolipids was studied using the chicken embryo (*in ovo*) model. The results obtained demonstrated that rhamnolipids produced by *Pseudomonas aeruginosa* #112 and commercial rhamnolipids (RL-90) significantly reduced blood glucose levels of chicken embryos on embryonic day 11 (from 135 ± 11 mg/dL to 94–107 mg/dL). These values were similar to those achieved with human insulin (104 ± 20 mg/dL) and the rapid-acting human insulin analog FIASP^®^ (95 ± 18 mg/dL). It was also verified that rhamnolipids did not have a negative effect on chicken embryo development at the concentrations tested. Regarding the molecular mechanisms involved in the decrease in blood glucose levels, for both insulins, a reduction in the expression of genes encoding the glucose transporter 2 (*glut2*) and the gluconeogenic enzymes phosphoenolpyruvate carboxykinase 2 and fructose-1,6-biphosphatase 1 was observed. However, in the case of rhamnolipids, only a reduction in the expression of *glut2* was observed. According to the results obtained, rhamnolipids are potential candidates for further studies on the development of new alternative treatments for diabetes symptoms.

## 1. Introduction

Diabetes mellitus, commonly referred to as diabetes, is a group of metabolic diseases that currently affects more than 580 million adults worldwide, and which incidence has increased in recent decades, mainly due to risk factors such as overweight and obesity, an increase in the consumption of processed and sugary foods, and a more sedentary lifestyle, but also as a result of population aging and environmental changes [1,2,3]. According to the last report from the International Diabetes Federation, it is estimated that the number of adults with diabetes will reach 853 million by 2050. Furthermore, the number of deaths caused by diabetes in 2024 is estimated at 3.4 million worldwide, 63% of them occurring in people over 60 years old [2]. Therefore, diabetes is considered a global health problem and belongs to the group of four non-communicable priority diseases. Diabetes also has a huge economic impact. It is estimated that in 2025, health expenditures due to diabetes exceeded one trillion USD worldwide [2].

Diabetes is a chronic disease characterized by elevated blood glucose levels (hyperglycemia). Pancreatic β-cells synthesize the hormone insulin, whose main function is to facilitate the absorption of glucose by the cells of peripheral tissues. High levels of glucose in the bloodstream stimulate insulin production. Insulin, transported by the bloodstream, binds to insulin receptors in the plasma membrane of hepatocytes, adipocytes, and skeletal muscle cells, promoting the transport of glucose from blood into those cells by activating glucose transporters (GLUTs), integral membrane proteins that allow glucose exchange between the extracellular space and the cytosol through facilitated diffusion. Once inside the cells, glucose is stored or transformed into energy. In individuals with type 1 diabetes mellitus (T1DM), hyperglycemia occurs mainly due to a defective insulin secretion by pancreatic β-cells, whereas in those suffering type 2 diabetes mellitus (T2DM), hyperglycemia is mainly due to insulin resistance of peripheral tissue cells (mainly from skeletal muscle, liver, and adipose tissue), i.e., they do not respond in a normal way to insulin. Hyperglycemia causes dysfunctions in the metabolism of carbohydrates, lipids, and proteins, which in turn alter the normal function of different organs [4]. Cardiovascular diseases (e.g., hypertension, chronic coronary disease, heart attack, stroke), nephropathy, retinopathy (with eventual vision loss and even blindness), diabetic foot syndrome, and lower-limb amputation are some of the diseases and complications associated with diabetes, besides the reduction in life expectancy [1,2,3,4].

Nowadays, a considerable number of pharmacological approaches are available for the treatment of diabetes, exogenous insulin injection being one of the most effective options to improve glycemic control. However, some of those therapies exhibit limitations or side effects, and for that reason, it is necessary to look for new alternatives [3,5,6,7]. Biosurfactants, surface-active compounds of biological origin, have been widely studied in recent years as environmentally friendly alternatives to synthetic surfactants for application in several fields, due to their remarkable properties [8,9]. Furthermore, microbial biosurfactants have been explored for biomedical applications, as some of them exhibit antitumor, antimicrobial, antioxidant, and immunomodulatory activities, among others [10,11,12]. In that sense, although the application of microbial biosurfactants for the treatment of diabetes symptoms and complications has been scarcely studied, previous works demonstrated the positive effect of the oral administration of lipopeptide biosurfactants on the blood glucose levels of diabetic rats and mice [13,14,15,16], opening the possibility of their potential application in this field.

Haselgrübler and co-workers [6] proposed the Gluc-hen’s egg test (Gluc-HET) for the screening of compounds with hypoglycemic activity, as an alternative to traditional mammalian models such as rodents. Domestic chicken (*Gallus gallus domesticus*) embryos share morphological, biochemical, and genetic similarities with mammalian embryos, representing a relevant animal model to perform studies in the fields of embryology, angiogenesis, genomics, teratogenesis, immunology, epigenetics, drug screening, or drug delivery, among others [17,18]. In the Gluc-HET model, the compounds under study are applied on the eggshell membrane of chicken embryos (usually at embryonic day 10 (E10) or 11 (E11)); those compounds subsequently diffuse to the chorioallantoic membrane (CAM), and due to its high vascularization, they are quickly incorporated in the bloodstream, being possible to observe their effect on blood glucose levels between one and three hours after their administration [6]. In chicken embryos, insulin is not detected in blood up to embryonic day 12 (E12), which avoids interference of insulin with the applied compounds on blood glucose levels. Furthermore, 10- and 11-day-old chicken embryos exhibit high glucose levels and are insulin sensitive, making them an optimum model for the screening of compounds with hypoglycemic activity, as demonstrated in previous works [5,6,19,20]. The Gluc-HET model exhibits several advantages when compared to traditional mammalian models such as rodents. It is relatively inexpensive, and its operation is fast and easy. Furthermore, experiments performed with chicken embryos up to the 11th day of embryonic development are considered non-animal assays from a legal point of view, due to the lack of a fully developed nervous system at that stage [6]; consequently, ethical approval is not required. However, although the general steps of glucose metabolism are highly conserved, the Gluc-HET model exhibits limitations (for instance, chickens lack GLUT4, which is a relevant glucose transporter in mammals), and for that reason, the results obtained using this model must be confirmed using well-established models, such as rodents [19,20].

In this work, the Gluc-HET model was used to study the effect of rhamnolipids (glycolipid biosurfactants) on blood glucose levels of chicken embryos for the first time, followed by the study of the expression of genes encoding different GLUTs and gluconeogenic enzymes in the liver of chicken embryos treated with rhamnolipids and insulin.

## 2. Materials and Methods

### 2.1. Chemicals

Rhamnolipid standard (RL-90, 90% purity) was purchased from Sigma-Aldrich (Saint Louis, MO, USA). Human insulin (>26 units/mg protein) was purchased from Hoffmann-La Roche AG (Basel, Switzerland). The rapid-acting human insulin analog (insulin aspart) FIASP^®^ (Novo Nordisk A/S, Bagsvaerd, Denmark) was offered by a diabetic patient.

### 2.2. Buffers and Solutions

Different buffers and solutions were evaluated in the *in ovo* assays. Hanks’ balanced salt solution (HBSS): NaCl 140 mM; glucose 6 mM; KCl 5 mM; NaHCO_3_ 4 mM; CaCl_2_ 1 mM; MgCl_2_ 6 H_2_O 0.5 mM; MgSO_4_ 7 H_2_O 0.4 mM; KH_2_PO_4_ 0.4 mM; Na_2_HPO_4_ 0.3 mM. Phosphate-buffered saline (PBS): NaCl 137 mM; KCl 2.7 mM; Na_2_HPO_4_ 10 mM; KH_2_PO_4_ 1.8 mM. Sodium chloride 0.72% (*w*/*v*): NaCl 123 mM. Tris-HCl: tris(hydroxymethyl)aminomethane 10 mM. The pH of PBS and Tris-HCl was adjusted to 7.4, whereas the pH of HBSS and NaCl 0.72% was not adjusted.

### 2.3. Rhamnolipids Production and Purification

Rhamnolipid production and purification were performed as described in our previous work [9]. *Pseudomonas aeruginosa* #112 was used for rhamnolipids production in flasks, using the culture media Luria–Bertani (LB) and corn steep liquor-molasses (CSLM). Rhamnolipids purification from the cell-free supernatants was performed through adsorption chromatography using Amberlite^®^ XAD^®^-2 columns (Sigma-Aldrich, Saint Louis, MO, USA). The presence of rhamnolipids was followed through the production and purification processes through thin-layer chromatography (TLC), using standard rhamnolipids (RL-90) as reference, and through surface tension measurement, using a KRÜSS K6 tensiometer (KRÜSS GmbH, Hamburg, Germany), as described elsewhere [8]. Purified freeze-dried rhamnolipids were used to calculate the critical micelle concentration (CMC), according to Correia et al. [8], and were subsequently stored at −20 °C until use. A detailed description of the methodologies used for rhamnolipid production and purification is provided in the Supplementary Material file.

### 2.4. In Ovo Assays

**Figure 1:**
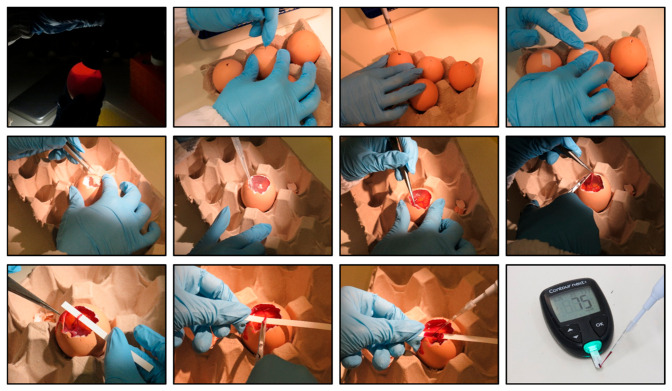
Main steps of the Gluc-HET model assays.

The methodology used to study the potential hypoglycemic activity of rhamnolipids was the Gluc-HET test proposed by Haselgrübler and co-workers [6]. Fertilized chicken eggs were obtained from a local breeder (Pintobar, Amares, Portugal) and stored at 16 °C (no longer than 10 days after being laid) until performing the assays. To allow the development of the chicken embryos, the eggs were incubated at 38 °C, with a humidity of around 45%, for 11 days. On the 11th day of incubation, to verify if the eggs were fertilized and the embryos developed as expected, before their manipulation, they were checked using the candling technique (Figure 1). This allows the selection of eggs containing embryos in the appropriate stage of development, as well as to locate the air bladder, which will be necessary for further steps. Once the appropriate eggs were selected, the first step consisted of making a small hole in the eggshell, in the region corresponding to the air bladder, which was used to place 200 µL of the corresponding test solution on the eggshell membrane, using a micropipette. After that, the hole was closed with adhesive tape, and the eggs were incubated again at the same conditions for 2 or 3 h, to allow the diffusion of the compounds injected through the eggshell membrane, achieving the CAM, and subsequently the bloodstream. After the incubation time, the eggshell above the air bladder was carefully removed using a micro-scissor and surgical forceps, and the exposed eggshell membrane was hydrated with 2–3 mL of PBS. Subsequently, the excess of PBS was discarded, and the eggshell membrane was carefully removed with the aid of a microscissor and surgical forceps to expose the CAM. Finally, the CAM was carefully cut using a microscissor to gain access to the chicken embryo. At this point, a suitable blood vessel was chosen for blood collection, which was carefully isolated and placed on a pH strip. After drying the area around the vessel with filter paper (to avoid blood dilution), the vessel was cut using a microscissor, and about 10 µL of blood was collected using a micropipette. Finally, glucose concentration was immediately measured using the Contour^®^NEXT blood glucose monitoring kit (Ascensia Diabetes Care Holdings AG, Basel, Switzerland). This device is used by individuals with diabetes to measure blood glucose levels; its detection range is between 20 and 600 mg glucose/dL, with a minimum sample volume of 0.6 µL. Human insulin and the insulin analog FIASP^®^ were used as positive controls. Blood glucose levels were also measured in embryos from eggs without manipulation (untreated eggs), and in embryos from drilled eggs where no solutions were injected, but that were incubated as the treated eggs (sham control).

### 2.5. Toxicity Tests

To evaluate the toxicity of the rhamnolipids under study, chicken embryos at E11 were treated with rhamnolipid solutions, as described in Section 2.4, and incubated under the same conditions, but in this case for 24 h. Subsequently, the eggs were carefully opened, and the embryos were checked for vitality, potential injuries, and delays in development. Eggs treated with the corresponding buffer under the same conditions were used as a control. Six embryos were analyzed for each treatment.

### 2.6. Ethical Statement

*In ovo* assays were performed within the first two-thirds of chicken embryonic development, and therefore do not require ethical approval, according to the European Parliament Directive 2010/63/EU of 22 September 2010, and the Portuguese Directive 113/2013 of 7 August 2013 on the protection of animals used for scientific purposes.

### 2.7. RNA Extraction and Gene Expression Assays

Livers were collected from chicken embryos after blood recovery (at the end of the Gluc-HET assays). Total RNA was extracted from livers using the NZY Total RNA Isolation Kit (NZYtech, Lisbon, Portugal), following the manufacturer’s instructions, and stored at −80 °C. The concentration and purity of the RNA samples were determined by measuring the absorbance at 260/280 nm using a NanoDrop (ND-1000, Thermo Fisher Scientific Inc., Waltham, MA, USA). Subsequently, 1.2 μg of RNA was treated with RNase-free DNase I (Thermo Fisher Scientific Inc., Waltham, MA, USA) and reverse transcribed into cDNA using the GRS cDNA Synthesis kit (GRiSP Lda., Porto, Portugal), following the manufacturers’ instructions.

Real-Time PCR (qPCR) assays were performed in a CFX Duet thermal cycler (Bio-Rad, Hercules, CA, USA). Gene-specific primers synthesized by Invitrogen (Thermo Fisher Scientific Inc., Waltham, MA, USA) were used to analyze the expression of the different genes under study (Table 1). Each reaction contained the optimized concentrations of cDNA and primers, and 5 µL of Xpert Fast SYBR (GRiSP Lda., Porto, Portugal), in a final volume of 10 µL. Subsequently, they were incubated at 95 °C for 2 min, followed by 40 cycles of 5 s at 95 °C, and 20 s at 60 °C. A melting curve (65 to 95 °C, 1 °C/min) was generated at the end of the reaction to assess the specificity of the amplification and discard the presence of primer dimers. *β-actin* and *18S* rRNA genes were used to normalize the expression of the target genes. Appropriate controls (no template control, no reverse transcriptase control, and negative control) were performed. The efficiency (*E*) of each set of primers was calculated by the CFX Maestro Software (version 2.3), using a calibration curve obtained using 10-fold serial dilutions of cDNA from the control assays as a template, and the thermal cycling conditions described above. Relative expression was calculated with the mathematical models proposed by Livak and Schmittgen [21] using the threshold cycle (*C_t_*) values obtained. Six independent samples of each condition were analyzed in duplicate.

### 2.8. Statistical Analysis

Data were expressed as the mean ± standard deviation of different numbers of replicates, depending on the assay. Results from the *in ovo* assays and gene expression were analyzed through one-way analysis of variance (ANOVA) followed by Tukey’s multiple comparisons test, at a significance level of 0.05, using the software GraphPad Prism (10.4.2).

## 3. Results and Discussion

### 3.1. Rhamnolipids Characterization

Table 2 summarizes the surface tension and CMC values obtained for rhamnolipids produced by *P. aeruginosa* #112 and standard rhamnolipids (RL-90). Rhamnolipids produced by *P. aeruginosa* #112, both in LB and CSLM media (RL-#112-LB and RL-#112-CSLM, respectively), exhibited lower surface tension and CMC values when compared to RL-90 (Table 2), indicating a higher effectiveness and efficiency. The chemical characterization of those rhamnolipid mixtures, performed in our previous work [9], is also presented in Table 2. The main congeners present in RL-90, RL-#112-LB, and RL-#112-CSLM are the mono-rhamnolipid Rha–C_10_–C_10_ and the di-rhamnolipid Rha–Rha–C_10_–C_10_, although in different proportions (Table 2). Furthermore, whereas in RL-90 those were the only congeners detected, *P. aeruginosa* #112 produced eight and nine different rhamnolipid congeners when grown in CSLM and LB medium, respectively [9]. Considering all the congeners produced, RL-90 and RL-#112-CSLM contain a higher proportion of mono-rhamnolipids when compared to RL-#112-LB (Table 2).

### 3.2. Effect of Different Buffers and Solutions on Blood Glucose Levels

The methodology herein used, based on the Gluc-HET model proposed by Haselgrübler and co-workers [6] to study the hypoglycemic potential of plant extracts, consists of the injection of rhamnolipids, dissolved in an appropriate buffer, into the air bladder of embryonated eggs, in order to study their impact on the blood glucose levels of chicken embryos. However, according to the work from the same authors, who evaluated the effect of different buffers (PBS, HBSS, HEPES, and Krebs-Ringer-phosphate-HEPES) on blood glucose levels, the buffer itself can significantly influence that parameter [6]. For that reason, before starting the study, a screening was performed to select the buffer with the lowest effect on blood glucose levels.

**Figure 2:**
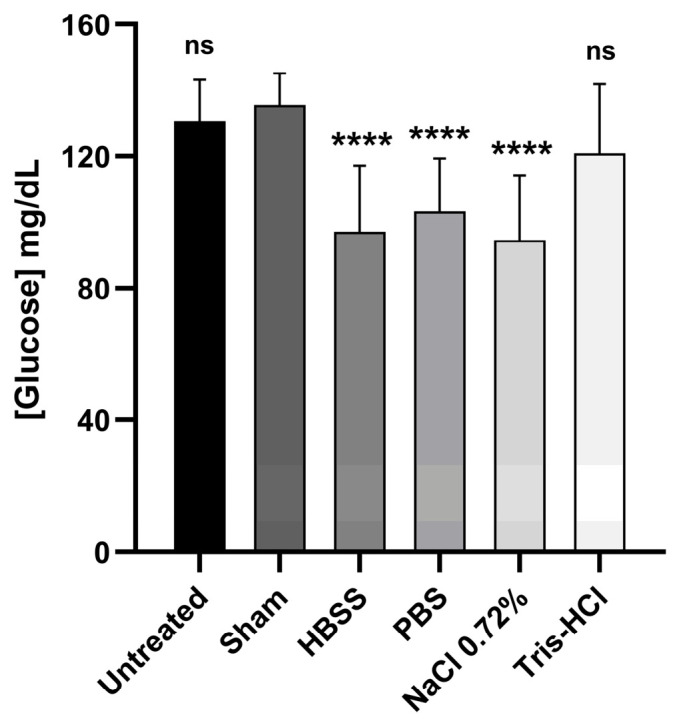
Effect of different buffers on blood glucose levels of chicken embryos. Eggs were incubated for 11 days and treated with 200 µL of the different buffers for 2 h. Results correspond to the average of 16 (untreated eggs), 15 (sham control), 20 (HBSS), 20 (PBS), 14 (NaCl 0.72%), and 31 (Tris-HCl) assays, and error bars represent the standard deviation of the mean. ns: no significant differences (*p* > 0.05); ****: significant differences (*p* < 0.0001) when compared to the sham control. Statistical significance was determined through the one-way analysis of variance (ANOVA) followed by Tukey’s multiple comparisons test.

Before performing the experiments, it was necessary to define some parameters. Regarding the incubation time with the buffers, insulin, and rhamnolipid solutions, 2 h was selected according to Haselgrübler and co-workers [6], who observed that as the incubation time with some buffers increased from 1 to 3 h, the decrease observed in blood glucose levels also increased. Other authors incubated the eggs with the compounds under study for 1 h, as it was enough to allow their complete absorption and incorporation in the bloodstream [5,7,19,20]. Buffers, insulin, and rhamnolipid solutions were injected at room temperature, as according to Haselgrübler and co-workers [6], the temperature of the injected buffer (23–38 °C) did not have a significant effect on blood glucose levels. The injection volume was set at 200 µL, since according to Haselgrübler and co-workers [6], when the volume of buffer injected increased from 100 to 300 µL, a significant reduction in blood glucose levels was observed. The results obtained with the four buffers studied are presented in Figure 2.

As can be seen in Figure 2, the differences observed in blood glucose levels between the sham control (136 ± 9 mg/dL) and untreated embryos (131 ± 12 mg/dL) were not statistically significant (*p* > 0.05). However, the treatment of the embryos with the different buffers for 2 h reduced blood glucose levels to values between 95 and 121 mg/dL. Significant differences (*p* < 0.0001) in blood glucose levels were observed for all the buffers studied when compared to the sham control, except for Tris-HCl, which resulted in the lowest reduction in blood glucose levels (121 ± 21 mg/dL) (Figure 2).

The percentages of reduction in blood glucose levels herein obtained with the different buffers (8–28%) are in agreement with those reported by Haselgrübler and co-workers [6], between 17 and 35% after 2 h of incubation when compared to untreated embryos. In that case, the buffer HBSS reduced blood glucose levels by 17%, and it was selected to perform the following studies [6]; however, in the present work, the same buffer lowered blood glucose levels by 26%. According to the results obtained, Tris-HCl was selected to dissolve the biosurfactants under study for their application in chicken embryos, due to its lower effect on blood glucose levels.

It is not clear why the treatment with certain buffers results in a significant decrease in blood glucose levels in chicken embryos. According to Haselgrübler and co-workers [6], this could be due to a dilution effect (which can explain why lower volumes of buffer have less effect), but also to some stress caused by the experimental process, which would promote a higher consumption of glucose and a consequent decrease in blood glucose levels. However, those hypotheses do not explain the differences observed between buffers with different compositions.

### 3.3. Effect of Insulin on Blood Glucose Levels

Once the most appropriate buffer was selected to perform the experiments, to validate the parameters previously selected, the effect of insulin on blood glucose levels in chicken embryos was studied. A rapid-acting human insulin analog (FIASP^®^), dissolved in Tris-HCl at two different concentrations (3 and 6 U/mL), was used, according to previous works [5,6,7,19]. The effect of human insulin, which can be considered an intermediate-acting insulin due to its slower activity, was also evaluated at the same concentrations. The decreases observed in blood glucose levels for both insulins after 2 h of treatment (between 1 and 8%) were not statistically significant (*p* > 0.05) when compared to Tris-HCl. However, other authors reported decreases in blood glucose levels ranging from 7 to 16% and 16 to 30% in similar assays, after 1 or 2 h of treatment, respectively, using similar concentrations of the rapid-acting human insulin analog NovoRapid^TM^ (Novo Nordisk A/S, Bagsvaerd, Denmark) [5,6,7,19]. The differences observed could be due to some variability in the experiments performed in different laboratories, but also to the different buffers used to solubilize the insulin (Tris-HCl *versus* HBSS).

**Figure 3:**
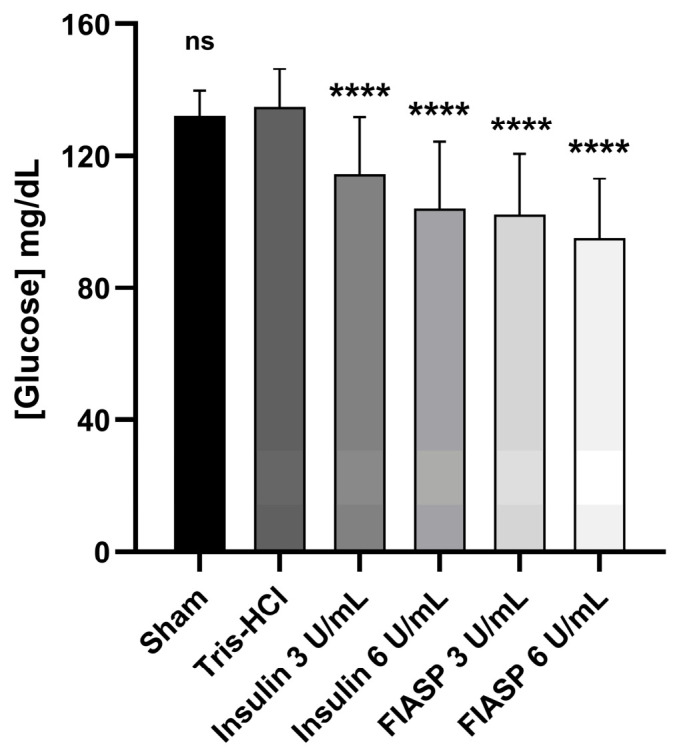
Effect of human insulin and a rapid-acting human insulin analog (FIASP^®^) dissolved in Tris-HCl at different concentrations (3 U/mL and 6 U/mL) on blood glucose levels of chicken embryos. Eggs were incubated for 11 days and treated with 200 µL of the different solutions for 3 h. Results correspond to the average of 7 (sham control), 57 (Tris-HCl), 20 (human insulin 3 U/mL), 26 (human insulin 6 U/mL), 24 (FIASP^®^ 3 U/mL), and 29 (FIASP^®^ 6 U/mL) assays, and error bars represent the standard deviation of the mean. ns: no significant differences (*p* > 0.05); ****: significant differences (*p* < 0.0001) when compared to Tris-HCl. Statistical significance was determined through one-way analysis of variance (ANOVA) followed by Tukey’s multiple comparisons test.

In order to study whether increasing the time of treatment with both insulins would result in a significant reduction in blood glucose levels, the same experiments described above were performed, but with an increased incubation time up to 3 h. At this point, it was necessary to check the effect of Tris-HCl on blood glucose levels for 3 h of treatment. As it can be seen from the results obtained (Figure 3), no significant differences (*p* > 0.05) were observed between chicken embryos corresponding to the sham control incubated for 3 h (132 ± 7 mg glucose/dL) and chicken embryos treated with Tris-HCl for 3 h (135 ± 11 mg/dL), validating the selection of this buffer to perform the experiments with an incubation time of 3 h.

Regarding the effect of insulin on blood glucose levels, for both insulins at both concentrations, the decrease observed after 3 h was higher when compared to 2 h of treatment, and those reductions, in all cases, were statistically significant (*p* < 0.0001) when compared to the control (Tris-HCl) (Figure 3). As expected, higher reductions in blood glucose levels were obtained for FIASP^®^ when compared to human insulin, due to its faster mechanism of action, and a dose effect was observed for both insulins (Figure 3). The highest decrease in blood glucose levels (95 ± 18 mg/dL) was obtained with the administration of FIASP^®^ (6 U/mL) (Figure 3). For the different insulin treatments, statistically significant differences were only observed between the treatment with FIASP^®^ at 6 U/mL and human insulin at 3 U/mL (*p* < 0.001). According to the results obtained, to provide enough time to allow the absorption of rhamnolipids, the incubation time was increased from 2 to 3 h in the following experiments.

### 3.4. Effect of Rhamnolipids on Blood Glucose Levels

**Figure 4:**
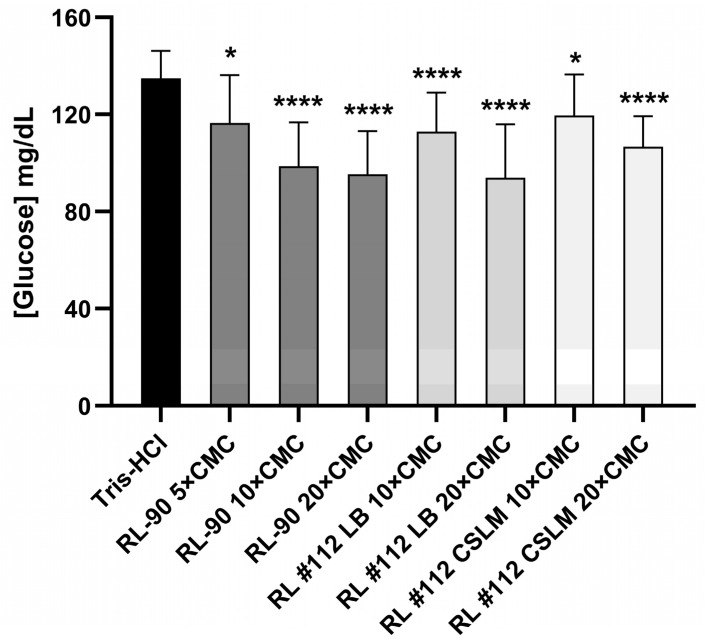
Effect of commercial rhamnolipids (RL-90), and rhamnolipids produced by *Pseudomonas aeruginosa* #112 in LB (RL-#112-LB) and CSLM medium (RL-#112-CSLM), dissolved in Tris-HCl at different concentrations, on blood glucose levels of chicken embryos. Eggs were incubated for 11 days and treated with 200 µL of the different solutions for 3 h. Results correspond to the average of 57 (Tris-HCl), 13 (RL-90 20 × CMC), 23 (RL-90 10 × CMC), 12 (RL-90 5 × CMC), 23 (RL-#112-LB 20 × CMC), 23 (RL-#112-LB 10 × CMC), 13 (RL-#112-CSLM 20 × CMC), and 15 (RL-#112-CSLM 10 × CMC) assays, and error bars represent the standard deviation of the mean. *: significant differences (*p* < 0.05); ****: significant differences (*p* < 0.0001) when compared to Tris-HCl. Statistical significance was determined through one-way analysis of variance (ANOVA) followed by Tukey’s multiple comparisons test.

In order to study the hypoglycemic activity of rhamnolipids in chicken embryos, commercial rhamnolipids (RL-90) and rhamnolipids produced by *P. aeruginosa* #112 were evaluated at different concentrations, selected according to their CMC values (between 5 and 20 times the CMC). As can be seen in Figure 4, at the highest concentration tested (20 × CMC), RL-90 and rhamnolipids produced by *P. aeruginosa* #112 in both culture media significantly (*p* < 0.0001) reduced blood glucose levels when compared to Tris-HCl. The same was observed for RL-90 and RL-#112-LB at a concentration corresponding to 10 × CMC; however, in the case of rhamnolipids produced in CSLM medium, at that concentration, the reduction observed in blood glucose levels was less significant (*p* < 0.05) (Figure 4).

**Figure 5:**
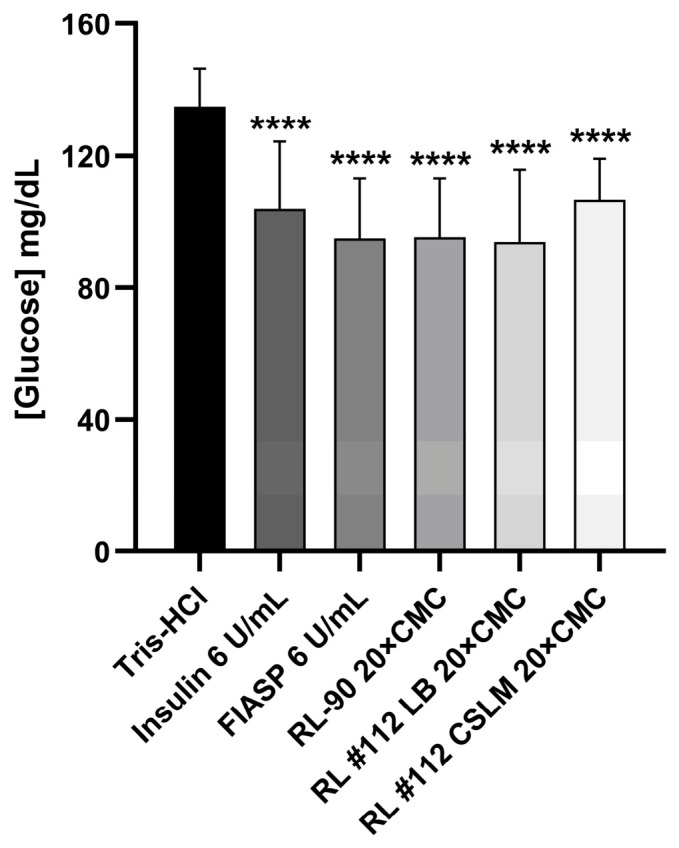
Effect of human insulin, FIASP^®^, commercial rhamnolipids (RL-90), and rhamnolipids produced by *Pseudomonas aeruginosa* #112 in LB (RL-#112-LB) and CSLM medium (RL-#112-CSLM), dissolved in Tris-HCl at different concentrations, on blood glucose levels of chicken embryos. Eggs were incubated for 11 days and treated with 200 µL of the different solutions for 3 h. Results correspond to the average of 57 (Tris-HCl), 26 (human insulin 6 U/mL), 29 (FIASP^®^ 6 U/mL), 13 (RL-90 20 × CMC), 23 (RL-#112-LB 20 × CMC), and 13 (RL-#112-CSLM 20 × CMC) assays, and error bars represent the standard deviation of the mean. ****: significant differences (*p* < 0.0001). Statistical significance was determined through one-way analysis of variance (ANOVA) followed by Tukey’s multiple comparisons test.

The effect of rhamnolipids on blood glucose levels of chicken embryos was compared to the effect of human insulin and FIASP^®^ at the highest concentration tested (6 U/mL). For the three rhamnolipid mixtures at a concentration of 20 × CMC, no significant differences (*p* > 0.05) were observed in blood glucose levels when compared to human insulin (6 U/mL) and FIASP^®^ (6 U/mL) (Figure 5), highlighting the hypoglycemic activity and the fast mechanism of action of these biosurfactants. It is important to highlight that, in the case of RL-90 and RL-#112-LB, blood glucose levels were reduced to 95 ± 17 and 94 ± 21 mg/dL, respectively, similar to the values achieved with FIASP^®^ 6 U/mL (95 ± 18 mg/dL), and lower than those achieved with human insulin (104 ± 20 mg/dL).

Rhamnolipids produced by *P. aeruginosa* #112 in both culture media and RL-90 are quite similar regarding the main congeners present, although their relative percentages are different, as discussed in Section 3.1. However, despite the fact that RL-90 and RL-#112-CSLM contained a higher percentage of mono-rhamnolipids when compared to RL-#112-LB (Table 2), RL-90 and RL-#112-LB were more efficient in reducing blood glucose levels than RL-#112-CSLM, suggesting that the relative abundance of the different rhamnolipid congeners does not have a significant effect on the hypoglycemic activity of the rhamnolipid mixture.

The results obtained herein are quite relevant when compared to previous studies that used the same model. Extracts obtained from different plants, such as *Acacia catechu*, *Bellis perennis*, *Geum urbanum*, *Hippophae rhamnoides*, *Mentha spicata*, *Pulmonaria officinalis*, *Sapindus mukorossi*, *Saponaria officinalis*, or *Solidago* sp., reduced blood glucose levels by 4–30% in assays performed using the Gluc-HET model. Furthermore, the hypoglycemic effect of those extracts was, in most cases, lower when compared to the rapid-acting human insulin analog used as a control in those studies [5,6,7,19,20]. The results presented herein showed that rhamnolipids can reduce blood glucose levels of chicken embryos by 30%, similarly to the rapid-acting human insulin analog FIASP^®^ at a concentration of 6 U/mL.

Only a few works studied the potential application of biosurfactants for the mitigation of diabetes symptoms. Studies performed by Zouari and co-workers [13] demonstrated that the oral administration of a mixture of lipopeptide biosurfactants (surfactin, fengycin, and iturin) to alloxan-induced T1DM rats reduced blood glucose levels, exerted antilipidemic activity, and preserved the function of liver, kidneys, intestinal tissues, and pancreatic β-cells. Further studies performed by Chen and co-workers [14,15,16] demonstrated that surfactin reduced insulin resistance and lowered blood glucose levels in assays performed in vitro and in vivo. Surfactin significantly increased glucose consumption by insulin-resistant hepatocytes (IR-HepG2) and Caco-2 cells, by increasing the levels of the glucose transporter GLUT4 in their cytoplasmic membranes; furthermore, surfactin reduced the gluconeogenesis activity in IR-HepG2 cells [14]. In in vivo assays, the oral administration of surfactin to T2DM mice significantly reduced fasting blood glucose levels, due to the reduction in insulin resistance and the improvement of glucose metabolism in the liver. Furthermore, serum levels of triglycerides were reduced, and surfactin exhibited a protective effect on pancreatic β-cells, liver, and intestinal tissues of T2DM mice [14,15,16]. Additionally, the oral administration of surfactin to mice before T2DM induction (through a high-fat diet combined with streptozotocin treatment) delayed T2DM development and mitigated its symptoms [15]. Despite these promising results, it is not clear why lipopeptide biosurfactants exert those activities, and whether those positive effects could also be obtained with other biosurfactants.

### 3.5. Study of the Molecular Mechanisms Involved in the Reduction in Blood Glucose Levels in Chicken Embryos

In order to identify some of the molecular mechanisms involved in the reduction in blood glucose levels, the expression of selected genes in the liver of chicken embryos treated with insulin (human insulin and FIASP^®^) and rhamnolipids (RL-90 and RL-#112-LB) was studied. Five genes related to glucose metabolism were selected according to previous works [17,22,23]: three genes encoding glucose transporters (*glut1*, *glut2*, and *glut3*), and two genes encoding gluconeogenesis key enzymes (phosphoenolpyruvate carboxykinase 2 (*pck2*) and fructose-1,6-biphosphatase 1 (*fbp1*)) (Table 1).

**Figure 6:**
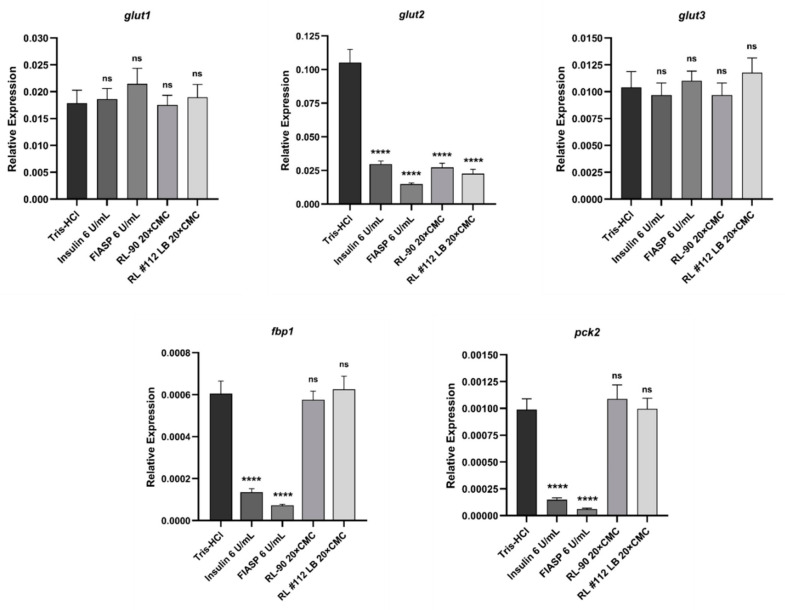
Relative expression of the genes *glut1*, *glut2*, *glut3*, *pck2*, and *fbp1* in the liver of chicken embryos treated with human insulin (6 U/mL), the rapid-acting human insulin analog FIASP^®^ (6 U/mL), commercial rhamnolipids (RL-90 20 × CMC), and rhamnolipids produced by *Pseudomonas aeruginosa* #112 in LB (RL-#112-LB 20 × CMC), when compared to the control (Tris-HCl). Results correspond to six independent samples analyzed in duplicate, and error bars represent the standard deviation of the mean. The *β-actin* and *18S* rRNA genes were used as internal controls to normalize the expression data. ns: no significant differences (*p* > 0.05); ****: significant differences (*p* < 0.0001) when compared to Tris-HCl. Statistical significance was determined through one-way analysis of variance (ANOVA) followed by Tukey’s multiple comparisons test.

The results obtained for the expression of the five genes are presented in Figure 6. The expression of *glut1* and *glut3* was not affected by the treatment with insulin or rhamnolipids. However, the expression of *glut2* was significantly reduced (*p* < 0.0001) in the liver of chicken embryos treated with both insulins and both rhamnolipid mixtures when compared to the control (Tris-HCl), suggesting a relationship between the reduction in the hepatic transcript levels of the glucose transporter GLUT2 and the observed reduction in blood glucose levels. There are several GLUTs, and their distribution and regulation are species-, tissue-, and time-specific [14,23,24]. Although GLUTs usually transport glucose from the extracellular space to the cells, GLUT2 also exports glucose produced by hepatocytes through gluconeogenesis to the bloodstream [24]. Consequently, the reduction in the hepatic levels of GLUT2 can contribute to the reduction observed in blood glucose levels in chicken embryos treated with rhamnolipids and insulin.

Regarding the effect on the expression of genes encoding the gluconeogenic enzymes PCK2 and FBP1, a significant effect (*p* < 0.0001) was only observed in the livers of chicken embryos treated with both insulins, whereas no significant effect was observed in the livers of chicken embryos treated with rhamnolipids. The reduction in the levels of PCK2 and FBP1 is in agreement with a reduction in blood glucose levels, due to a decrease in the gluconeogenesis activity [23]. Although that effect was observed in the liver of chicken embryos treated with insulin, it was not observed for rhamnolipids, meaning that other mechanisms may be involved in the reduction in blood glucose levels in this case.

Few studies have investigated the mechanism of action of exogenous insulin on glucose metabolism in chicken embryos. Frannssens and co-workers [23] studied the effect of exogenous insulin on blood glucose levels in chicken embryos on embryonic day 16 (E16) and 18 (E18). In that case, bovine insulin dissolved in NaCl 0.9% (0.5 U insulin/embryo) was injected directly into a blood vessel of the CAM. Blood glucose levels were reduced between four and five times in E16 and E18 embryos when compared to non-treated embryos, with the highest reduction achieved 7 h after insulin injection, with a more evident effect in E16 embryos. In order to study the mechanisms involved, the effect of insulin on the expression of genes encoding GLUT1, GLUT2, GLUT3, GLUT8, GLUT9, and GLUT12 in the liver of E16 and E18 embryos was studied. Among them, only GLUT2 was significantly affected. The expression of *glut2* in hepatocytes was reduced two times in insulin-treated E16 embryos 7 h after insulin administration when compared to non-treated embryos; however, that effect was not observed in E18 embryos. The effect of insulin treatment on the expression of genes encoding gluconeogenesis key enzymes (PCK1, PCK2, and FBP1) in hepatocytes of E16 and E18 embryos was also studied. In this case, PCK1 was not affected, but insulin treatment significantly reduced the levels of *pck2* and *fbp1* transcripts 7 h after insulin injection when compared to the non-treated embryos, with that effect being more significant in E16 embryos. These results confirmed that key enzymes and transporters involved in glucose metabolism are regulated by exogenous insulin in chicken embryos, although the effect depends on the embryonic stage. Insulin treatment reduced hepatic gluconeogenesis and, at the same time, reduced the efflux of glucose from hepatocytes to the bloodstream, contributing to reducing blood glucose levels [23]. These results are in agreement with those obtained in the present work for E11 chicken embryos treated with insulin, whereas in the case of chicken embryos treated with rhamnolipids, only a significant effect in the expression of *glut2* was observed.

### 3.6. Toxicity Assays

**Figure 7:**
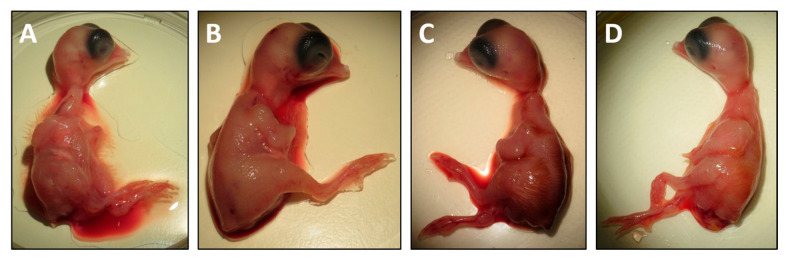
Appearance of chicken embryos treated for 24 h with Tris-HCl (**A**), commercial rhamnolipids (RL-90) (**B**), and rhamnolipids produced by *Pseudomonas aeruginosa* #112 in LB (**C**) and CSLM (**D**). Rhamnolipids were dissolved in Tris-HCl at a concentration corresponding to 20 × CMC.

In order to check the effect of rhamnolipids on chicken embryos’ vitality and development, 200 µL of the different rhamnolipid solutions, at a concentration corresponding to 20 × CMC, were injected into embryonated eggs at E11, which were incubated as in the previous assays, but in this case for 24 h. Subsequently, the eggs were opened, and the embryos were checked for vitality and potential delays in development. Illustrative images of chicken embryos after treatment are shown in Figure 7. All chicken embryos treated with the different rhamnolipids were alive after 24 h of treatment and exhibited a similar size and appearance to those treated with Tris-HCl (control). Accordingly, it can be concluded that those rhamnolipids did not have a detrimental effect on chicken embryo development in the conditions herein studied.

Toxicity studies for biosurfactants using the *in ovo* model are scarce. Behzadnia and co-workers [10] studied the inhibitory activity of the biosurfactant produced by *Lactobacillus plantarum* ATCC 8014 against the virus responsible for Newcastle disease using the *in ovo* model. The toxicity assays performed simultaneously demonstrated that the biosurfactant did not have detrimental effects in E9 chicken embryos, at concentrations between 2 and 30 mg/mL, after 48 h of treatment [10].

## 4. Conclusions

The results obtained demonstrated, for the first time, the hypoglycemic activity of rhamnolipids in chicken embryos. Commercial rhamnolipids and rhamnolipids produced by *P. aeruginosa* #112 in LB medium reduced blood glucose levels by 30%, similar to the results achieved with the rapid-acting human insulin analog FIASP^®^. The hypoglycemic activity of rhamnolipids can be partially explained by a decrease in GLUT2 levels in the liver of chicken embryos, which was also observed for insulin. Although the results obtained are promising, further studies are necessary to confirm the hypoglycemic activity of rhamnolipids in other animal models and to validate the results obtained in chicken embryos.

## Figures and Tables

**Table 1:** Primers used to study gene expression through qPCR. *E* (%): primer efficiency. Tm (°C): primer melting temperature.

Target Gene	Nucleotide Sequence (5′→3′)	*E* (%)	Tm (°C)	Ref.
*glut1*	Fw: GCAGTTCGGCTACAACACCG	103	61.90	[17]
	Rv: ATCAGCATGGAGTTACGCCG		60.53	
*glut2*	Fw: CAGGAACGTTGGTCCTCTCC	95	60.04	[22]
	Rv: GCGCCCATAGTGTGCTTCTA		60.18	
*glut3*	Fw: GTACCGTTCGGGTTCCGTTAG	96	60.73	[17]
	Rv: AATGGCAGCAACAGAAACAGC		60.27	
*pck2*	Fw: GGCCGAGCACATGCTGATTT	97	61.66	[23]
	Rv: CCGCCATGTAACGCTTCTCA		60.74	
*fbp1*	Fw: TGCTGCGGTCACCGAGTATCTCA	94	65.70	[23]
	Rv: TCATAGAGCAGTCTCAGCTTCCCTT		63.10	
*β* *-* *actin*	Fw: CTTCTAAACCGGACTGTTACCA	104	57.74	[17]
	Rv: AAACAAATAAAGCCATGCCAATCT		58.19	
*18S*	Fw: TCTTTCTCGATTCCGTGGGT	99	58.74	[17]
	Rv: AACGCCACTTGTCCCTCTAC		59.68	

**Table 2:** Minimum surface tension (ST, mN/m) and critical micelle concentration values (CMC, mg/L) obtained for purified rhamnolipids (RL) produced by *Pseudomonas aeruginosa* #112 in LB (RL-#112-LB) and CSLM (RL-#112-CSLM), and standard rhamnolipids (RL-90), dissolved in PBS. Congeners composition according to Gudiña and co-workers [9].

RL	ST (mN/m)	CMC (mg/L)	Main Congeners	Mono-RL: Di-RL Ratio
RL-#112-LB	32.5 ± 0.3	57	Rha–C_10_–C_10_: 27% Rha–Rha–C_10_–C_10_: 35%	0.84
RL-#112-CSLM	30.9 ± 0.1	56	Rha–C_10_–C_10_: 45% Rha–Rha–C_10_–C_10_: 26%	1.60
RL-90	33.4 ± 0.2	89	Rha–C_10_–C_10_: 68% Rha–Rha–C_10_–C_10_: 32%	2.12

## Data Availability

The original contributions presented in the study are included in the article; further inquiries can be directed to the corresponding authors.

## Supplementary Materials

The following supporting information can be downloaded at: https://www.mdpi.com/article/10.3390/cimb48070664/s1.
