# Supplementary material for: Study of the Hypoglycemic Activity of Rhamnolipids Using the In Ovo Model
Source: Curr Issues Mol Biol. 2026 Jun 28;48(7):664. doi: 10.3390/cimb48070664 (PMC13406233; doi:10.3390/cimb48070664)
Supplement: Supplementary file 1 [file cimb-48-00664-s001.zip › cimb-4395323-supplementary.pdf]

Supplementary Material

Rhamnolipids Production and Purification

Rhamnolipids used in this work were produced using *Pseudomonas aeruginosa* #112, according to Gudiña and co-workers [9]. Two different culture media were used for rhamnolipid production: Luria Bertani (LB) and Corn Steep Liquor-Molasses (CSLM). The composition of LB medium was: NaCl 10 g/L; tryptone 10 g/L; yeast extract 5 g/L. The pH was adjusted to 7.0 using a 1 M NaOH solution. The composition of CSLM was: Corn Steep Liquor (CSL) 10% (v/v); sugarcane molasses 10% (w/v). CSL was provided by COPAM: Companhia Portuguesa de Amidos, S.A. (Portugal). Suagrcane molasses was provided by RAR: Refinarias de Açúcar Reunidas, S.A. (Portugal). Due to the acidic pH of CSLM medium (about 3), the pH was adjusted to 7 using a combination of NaOH pellets and a 1 M NaOH solution. Both media were autoclaved at 121°C for 15 minutes.

Rhamnolipid production was performed in 500 mL flasks containing 200 mL of LB or CSLM. Each flask was inoculated with 1% (v/v) of a pre-culture of *P. aeruginosa* #112 grown overnight in LB medium at 37°C and 200 rpm. After inoculation, the cultures were incubated at 37°C and 200 rpm for 48 h (LB) or 96 h (CSLM). Rhamnolipids production was assessed by measuring the surface tension of cell-free supernatants of samples taken every 24 h, as described below. Triplicate assays were performed for each culture medium, in batch mode.

Rhamnolipids produced in LB and CSLM were recovered from the cell-free supernatants obtained at the end of the production process. First, the culture media were centrifuged at 9000 rpm and 25°C for 30 minutes to remove the cells. Subsequently, the produced rhamnolipids were recovered from the cell-free supernatants through adsorption chromatography using the polystyrene resin Amberlite® XAD®-2 (particle size 20–60 mesh; mean pore size 90 A°; Sigma–Aldrich Co., USA), following the methodology described by Correia et al. [8]. The resin contained in a 250 mL glass column was equilibrated with 300 mL of 0.1 M potassium phosphate buffer (pH 6.1) at a constant flow rate of 3 mL/min, to activate its hydrophobic domains. Subsequently, the cell-free supernatants (100 mL) were adjusted to pH 6.1 and introduced into the column at the same flow rate. After that, the column was washed with 500 mL of demineralized water to remove the components that were not adsorbed to the resin. Finally, the rhamnolipids adsorbed to the resin were eluted with 300 mL of methanol. Subsequently, the methanol was allowed to evaporate in a fume hood, and the rhamnolipids recovered were dissolved in a small amount of demineralized water, freeze-dried, and stored at –20 °C until further use.

The presence of rhamnolipids was verified through thin layer chromatography (TLC). Briefly, samples of the different fractions recovered were spotted onto silica gel TLC plates (DC-Fertigfolien ALUGRAMR SIL G UV254, Macherey-Nagel GmbH & Co., Germany) that were developed using a solvent system consisting of chloroform:methanol:water (65:25:4, v/v/v). The isolated compounds on the TLC plate were located by spraying it with a solution containing orcinol (0.19% (w/v)) in 53% (w/v) sulphuric acid, followed by incubation at 105°C until the plots became visible. Commercial rhamnolipids (RL-90, 90% purity, Sigma-Aldrich Co., USA) were used as reference. The mono-rhamnolipid and di-rhamnolipid congeners present in the rhamnolipid mixtures exhibited a single spot, which Rf values were approximately 0.70 for mono-rhamnolipids and 0.47 for di-rhamnolipids.

Surface Tension Measurement

The surface tension of cell-free supernatants and biosurfactant solutions was measured using a Krüss K6 Tensiometer (KRÜSS GmbH) using the du Noüy ring method, at room temperature (20-25°C). Each sample was measured at least in triplicate.

Critical Micelle Concentration

The critical micelle concentrations (CMC) of commercial rhamnolipids, and rhamnolipids produced by *P. aeruginosa* #112 in LB and CSLM media were calculated by measuring the surface tension (as described above) of biosurfactant solutions prepared at different concentrations (between 15 and 1000 mg/L) in PBS buffer (NaCl 137 mM; KCl 2.7 mM; Na2HPO4 10 mM; KH2PO4 1.8 mM; pH 7.0). Each sample was measured at least in triplicate. The CMC was determined by plotting the surface tension as a function of the logarithm of biosurfactant concentration and was found at the point of intersection between the two lines that best fit through the pre- and post-CMC data.

References

[8] Correia, J.; Gudiña, E.J.; Lazar, Z.; Janek, T.; Teixeira, J.A. Cost-effective rhamnolipid production by *Burkholderia thailandensis* E264 using agro-industrial residues. *Appl. Microbiol. Biotechnol.* **2022**, *106*, 7477–7489. https://doi.org/10.1007/s00253-022-12225-1

[9] Gudiña, E.J.; Rodrigues, A.I.; Alves, E.; Domingues, M.R.; Teixeira, J.A.; Rodrigues, L.R. Bioconversion of agro-industrial by-products in rhamnolipids toward applications in enhanced oil recovery and bioremediation. *Bioresour. Technol.* **2015**, *177*, 87–93. https://doi.org/10.1016/j.biortech.2014.11.069
